# Underlying Mechanisms of *Bergenia* spp. to Treat Hepatocellular Carcinoma Using an Integrated Network Pharmacology and Molecular Docking Approach

**DOI:** 10.3390/ph16091239

**Published:** 2023-09-01

**Authors:** Shoukat Hussain, Ghulam Mustafa, Sibtain Ahmed, Mohammed Fahad Albeshr

**Affiliations:** 1 Department of Biochemistry, Government College University Faisalabad, Faisalabad 38000, Pakistan; 2 Scripps Institution of Oceanography, University of California San Diego, 9500 Gilman Drive, La Jolla, CA 92093, USA; 3 Department of Biochemistry, Bahauddin Zakariya University, Multan 60800, Pakistan; 4 Department of Zoology, College of Science, King Saud University, P.O. Box 2455, Riyadh 11451, Saudi Arabia

**Keywords:** hepatitis B virus, hepatitis C virus, (+)-catechin 3-gallate, β-sitosterol, KEGG, protein–protein interactions

## Abstract

Hepatocellular carcinoma (HCC) is the fifth most common and fatal cancer reported, representing 72.5% of malignancies around the world. The majority of HCC incidents have been associated with infections caused by hepatitis B and C viruses. Many first- and second-line conventional drugs, e.g., sorafenib, cabozantinib, or ramucirumab, have been used for the management of HCC. Despite different combinational therapies, there are still no defined biomarkers for an early stage diagnosis of HCC. The current study evaluated the potential of *Bergenia stracheyi*, *Bergenia ciliata*, *Bergenia pacumbis*, and *Bergenia purpurascens*, which belong to the family Saxifragaceae, to treat HCC using an integrated network pharmacology and molecular docking approach. Four active phytochemicals were selected based on oral bioavailability (OB) and drug likeness (DL) parameters. The criteria of phytochemical selection were set to OB > 30% and DL > 0.18. Similarly, the gene targets related to *Bergenia* spp. and the genes related to HCC were retrieved from different databases. The integration of these genes revealed 98 most common overlapping genes, which were mainly interrelated with HCC pathogenesis. Ultimately, the 98 *Bergenia*-HCC associated genes were used for protein–protein interaction (PPI), Kyoto Encyclopedia of Genes and Genomes (KEGG) pathway, and Gene Ontology (GO) enrichment analyses. Finally, the topological analysis revealed the top ten hub genes with maximum degree rank. From the top ten genes, STAT3, MAPK3, and SRC were selected due to their involvement in GO annotation and KEGG pathway. To confirm the network pharmacology results, molecular docking analysis was performed to target STAT3, MAPK3, and SRC receptor proteins. The phytochemical (+)-catechin 3-gallate exhibited a maximum binding score and strong residue interactions with the active amino acids of MAPK3-binding pockets (S-score: −10.2 kcal/mol), SRC (S-score: −8.9 kcal/mol), and STAT3 (S-score: −8.9 kcal/mol) as receptor proteins. (+)-Catechin 3-gallate and β-sitosterol induced a significant reduction in cell viability in HepG2 after 24 h of treatment in a dose-dependent manner. The results of this study explore the potential of (+)-catechin 3-gallate and β-sitosterol, which can be used in the future as potential drug candidates to suppress HCC.

## 1. Introduction

Hepatocellular carcinoma (HCC) or liver cancer is the second death-related cancer worldwide with late-stage diagnosis due to its severely fatal tumor [1]. Chronic liver infections such as hepatitis B, hepatitis C, alcoholic abuse, and metabolic disorders later progress into liver inflammation, liver fibrosis, and cirrhosis, which develop symptoms of HCC [2]. HCC is frequently multinodular upon diagnosis and it has a specific proclivity to develop inside the blood vessels and then infiltrate the portal or hepatic veins [3]. The prognosis of HCC is directly associated with tumor stage at the time of diagnosis. The early stage diagnosis of HCC is poorly reported, with a median of 5 years and a 7% survival rate [4]. HCC solely accounts for 75–85% of tumor cases in all liver cancer reports. In a report, the World Health Organization (WHO) predicted 1.3 million deaths by 2040 due to liver cancer [5]. The HCC-linked infection(s) could be recognized and decreased through inhibiting the development of HCC, which in turn increases the life span of the patients through early diagnosis with better combinations [6].

Different pathways including Wnt/ß-catenin pathway, phosphatidylinositol-3-kinase, MET receptor tyrosine kinase pathway, and Hedgehog (Hh) signaling pathway play a leading role in the proliferation of the tumor, which ultimately leads to HCC. Any mutational changes in the genetic makeup and signaling of the metabolic cascade mainly cause the onset of carcinogenesis [7]. Recently, against advanced HCC, sorafenib/lenvatinib and regorafenib/cabozantinib have been administrated as first-line and second-line drugs, respectively. Moreover, the combinational therapy of nivolumab and ipilimumab with pembrolizumab monotherapy has received FDA approval for the treatment of HCC [8]. Despite the combinational therapies and licensed drugs, the survival rate is limited due to the poor diagnosis and severe side effects of such drugs. Many other clinical trials including immune checkpoint inhibitors, chimeric antigen receptor T-cells, and dendritic cell vaccines are in the first stage of testing. Therefore, there is a dire need of drugs with no or fewer side effects for the treatment of HCC.

Plant-based herbal remedies have gained much interest in recent years for the treatment of various cancers. Plants are a natural source of biologically active compounds, which play a leading role as potential drug candidates for the treatment of multiple disorders and infections [9]. *Bergenia* is a genus of flowering plants and belongs to the Saxifragaceae family with about 35 known species. Different Ayurvedic formulations have used *Bergenia* species over the centuries to treat piles, kidney stones, bladder, and pulmonary infections [10]. Almost six species of this genus are present in Pakistan. These are mostly present in the northern areas of Pakistan, particularly in Kashmir or around it. They are present in a large number in temperate Himalayan regions at elevations from 2000 to 2700 m [11].

Network pharmacology is an elaborative domain that predicts the drug mechanism to treat different diseases, which is similar to Chinese medicine’s multitargeting functions. Network pharmacology is a multidimensional field in pharmacology, interpreting the network targeting and interrelation of plant phytochemicals to target enormous types of disorders [12]. In this study, the aim to treat HCC using phytochemical constituents of the *Bergenia* species was accomplished via network targeting of multidrug or multi-targeting approach. Using multi-therapeutics with the networking of targeted genes, biologically active phytochemicals have been used as leading drug candidates against key targeted genes with their role in different signaling mechanisms involved in the pathogenesis of HCC.

## 2. Results

### 2.1. Active Compounds of Bergenia spp.

A library was constructed which contained 16 phytochemicals from *B. ciliate*, 6 from *B. pacumbis*, 64 from *B. purpurascens*, and 61 from *B. stracheyi*. These phytochemicals were collected from different plant parts (e.g., roots, leaves, rhizome) reported in the literature and IMMPAT database. For network pharmacology analysis, only four compounds (i.e., β-sitosterol, cianidanol, (+)-catechin gallate, and leucocianidol were selected based on their pharmacokinetic criteria of drug likeness (DL > 0.18) and oral bioavailability (OB > 30%). In Table 1, different properties of the four compounds are given.

### 2.2. Target Prediction for Bergenia spp.

The *Bergenia* spp.-related gene targets were predicted from the SwissTargetPrediction and STITCH database. In total, 313 targets were predicted for the four phytochemicals of *Bergenia* spp. Then, 228 unique drug-related gene targets were predicted after removal of duplicates by aligning the UniprotKB protein IDs.

### 2.3. Target Prediction for Hepatocellular Carcinoma

**Figure 1:**
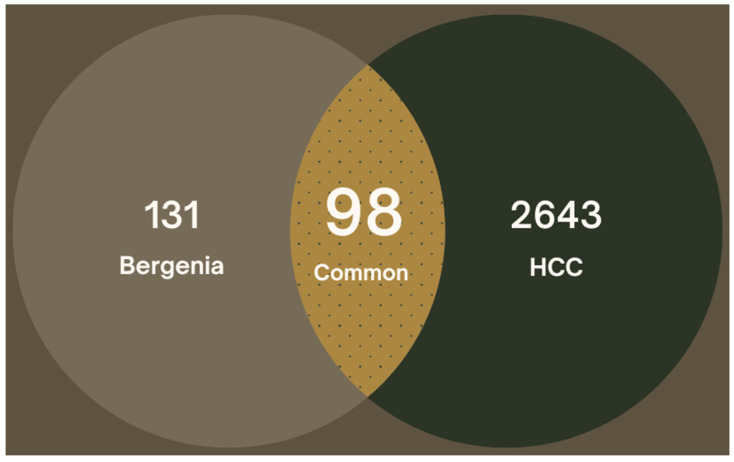
Venn illustration of potential targets.

A total of 3176 hepatocellular carcinoma-related gene targets were retrieved from GeneCard and DisGeNet databases. After removal of duplicates, a total of 2742 unique gene targets were used for further analysis. The intersection of *Bergenia* spp.-related targets and HCC-related predicted targets was performed for mapping of common overlapping gene targets. Out of these, a total of 98 targets were selected after interaction of drug-related gene targets and HCC-related gene targets (Figure 1).

### 2.4. Compound-Target Network

**Figure 2:**
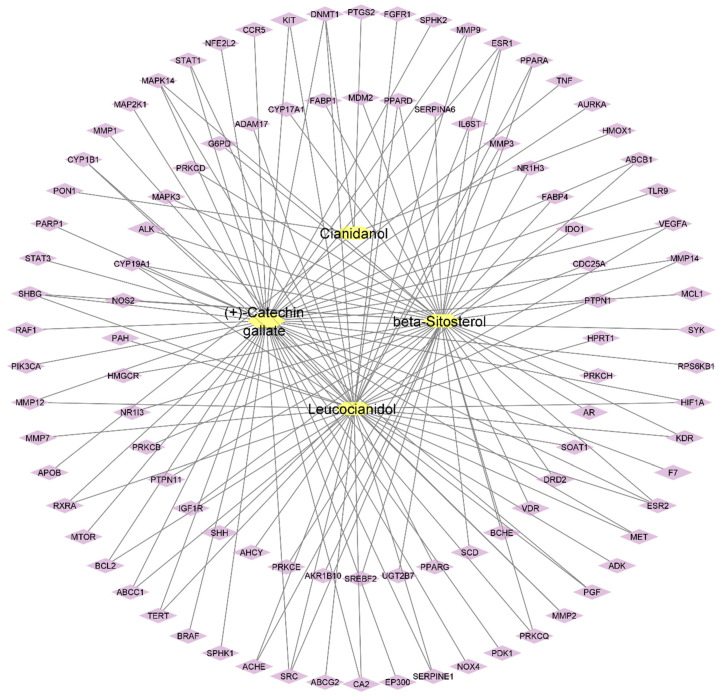
Compound–target network of active plant constituents and predicted targets (yellow: compounds, purple: target genes).

The Cytoscape software was used for compound–target network construction for four active plant constituents and 98 potential gene targets. A plug-in Network Analyzer was employed to calculate the topological parameters of the constructed network. The network has 102 nodes and 135 edges representing the active constituents and targeted genes, which are interrelated with lines (Figure 2). The topological analysis represented the network density of 0.026, network centralization of 0.448, and network heterogeneity of 2.694. Furthermore, the active phytochemicals were also categorized by degree method: (+)-catechin gallate (47), β-sitosterol (42), leucocianidol (41), and cianidanol (5), which represented the interactions with multiple targets. In network pharmacology, the network density is a quantitative measure, which is applied to characterize the interconnectedness of nodes (i.e., biological entities such as genes and proteins) within a network. A greater level of interactions between nodes is indicated by a high network density, which results in a more interconnected network. Within a biological network, such as drug–target interaction network or protein–protein interactional network, the network centralization is the degree to which some nodes play influential or important roles compared to other nodes. Similarly, network heterogeneity is considered as the diversity and complexity of interactions within biological networks, specifically, to reveal interactions between drugs, genes, proteins, and other molecules.

### 2.5. Protein–Protein Interactions (PPIs)

**Figure 3:**
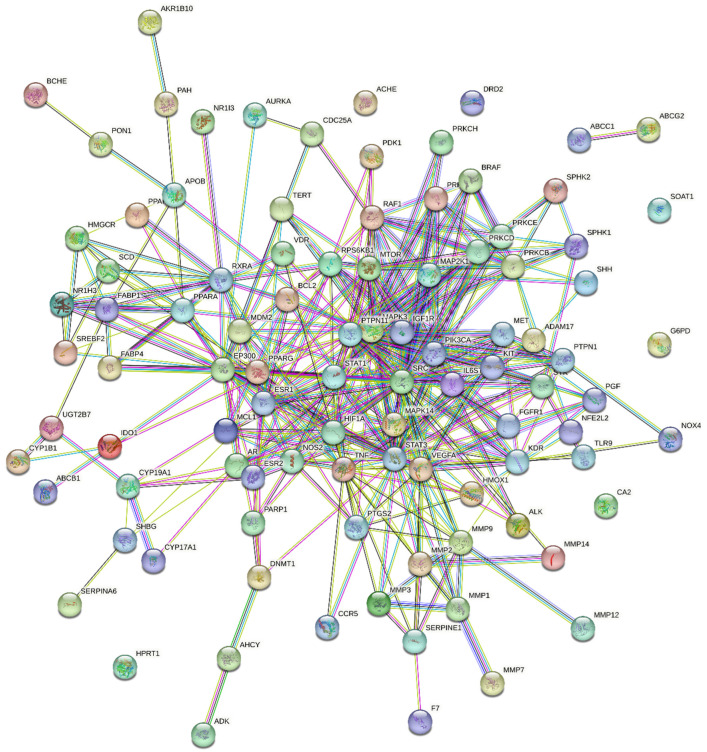
Protein–protein interaction diagram showing the interactions of 98 common target genes.

**Figure 4:**
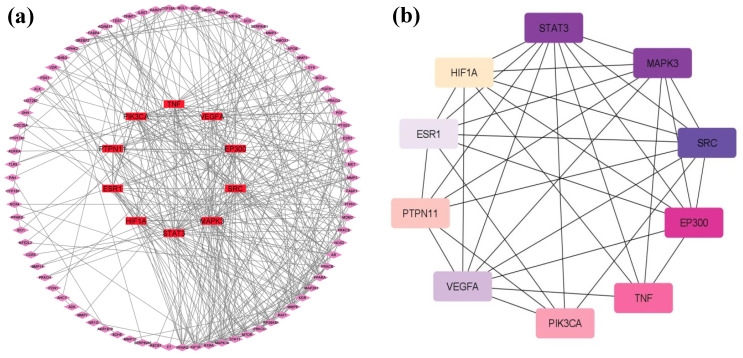
Protein–protein interactions showing: (**a**) interactions of 98 target genes with hub genes in the center (red); (**b**) hub genes with degree evaluation (the darker node is displaying higher rank).

The 98 potential targets were imported into STRING 11.5 for protein–protein interactions with a high confidence score of 0.700 and by selecting *H. sapiens* as the default organism (Figure 3). In the constructed PPI network, there were 98 nodes, 377 edges with an average node degree of 7.69, and a PPI enrichment *p*-value of <1.0 × 10^−16^. The compound–target network of 98 target genes only displayed interactions with (+)-catechin gallate, β-sitosterol, and leucocianidol. Therefore, the phytochemical cianidanol was eliminated from further analysis. In addition, the network density of 0.094, network heterogeneity of 0.907, and network centralization of 0.329 were also calculated by the Network Analyzer tool of the software Cytoscape. The plug-in cytoHubba was also employed to examine the hub genes. The network degree is the number of interactions a node (i.e., compound, gene, protein) makes with other nodes within a biological network. A node with a higher network degree is considered as a hub node, as it shows significant control or influence on the functioning of the network. The degrees of the top targeted genes are represented in Table 2 and Figure 4.

### 2.6. Analysis of Gene Enrichment

**Figure 5:**
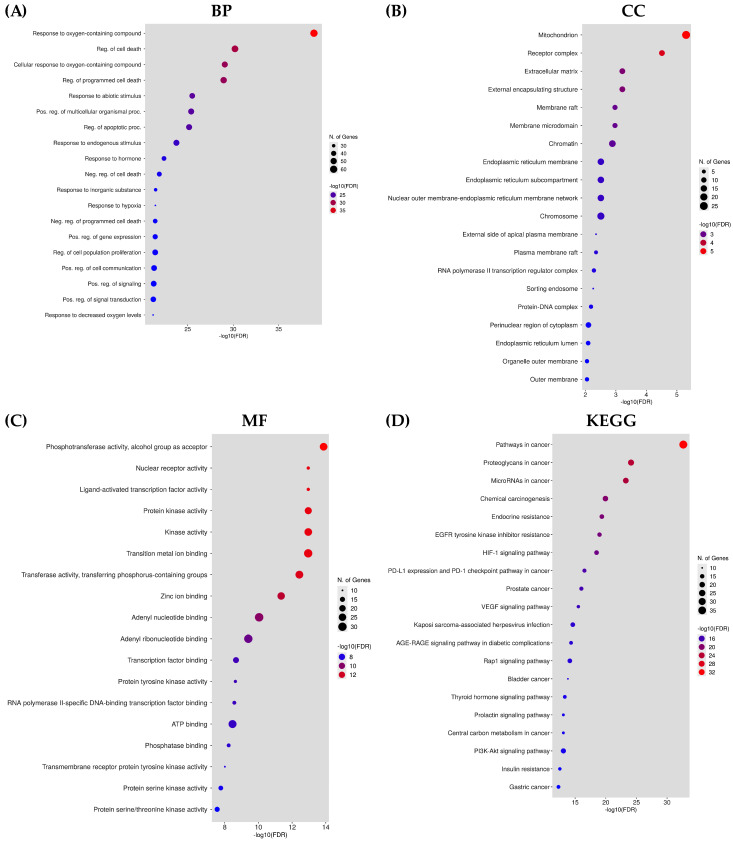
The illustration of functional annotation and enriched pathways in terms of HCC is shown by the bubble plot. (**A**) Gene ontology in terms of biological processes (BP). (**B**) Gene ontology in terms of cellular components (CC). (**C**) Gene ontology in terms of molecular function (MF). (**D**) KEGG pathway analysis.

The GO and KEGG enrichment analyses were performed through the DAVID database to predict the functional annotation and pathway enrichment associated with active plant constituents for the treatment of hepatocellular carcinoma. The GO annotation revealed 373 biological processes (BPs), 108 molecular functioning (MF), 40 cellular components (CCs), and 135 KEGG pathways. The top 20 terms in GO and KEGG analyses were identified as mainly involved in cancer pathways (Figure 5). The BPs mainly contain the cellular response toward oxygen-containing compounds, the regulation of programmed cell death, cell proliferation, and responses to stimuli, hormones, and hypoxia. The CC GO annotation was related to mitochondria, ER, receptor complex, chromosome, extracellular matrix, and membrane compartments. The MF was related to phosphotransferase activity, ligand-activated transcription factor activity, nuclear receptor activity, protein kinase activity, and ATP binding. The analysis of the KEGG pathway mainly pointed to the involvement of genes in pathways related to cancer, proteoglycans in cancer, microRNAs in cancer, EGFR tyrosine kinase inhibitor resistance, endocrine resistance, and prostate cancer. These pathways are all co-related directly or indirectly with the onset of hepatocellular carcinoma.

**Figure 6:**
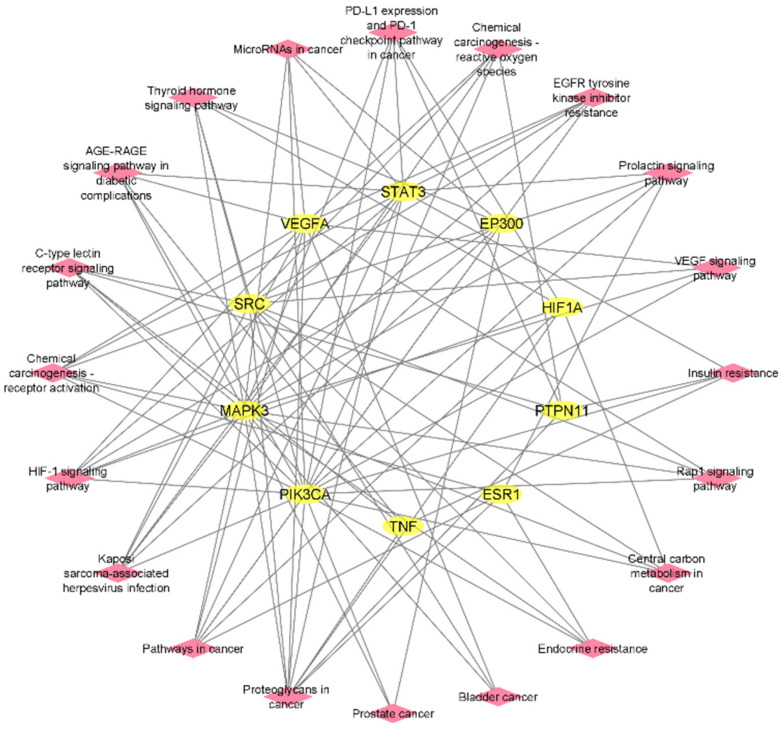
Target–pathway network of hub genes and 20 top enrichment analysis pathways (yellow: hub genes; pink: pathways).

The ShinyGO tool was used for interpretation and visualization of the top 20 selected pathways in the bar plot. The target–pathway network was constructed with the cystoscope software to fully understand the interrelation of targets with the associated signaling pathways (Figure 6).

### 2.7. Construction of Compound–Target–Pathway Network

**Figure 7:**
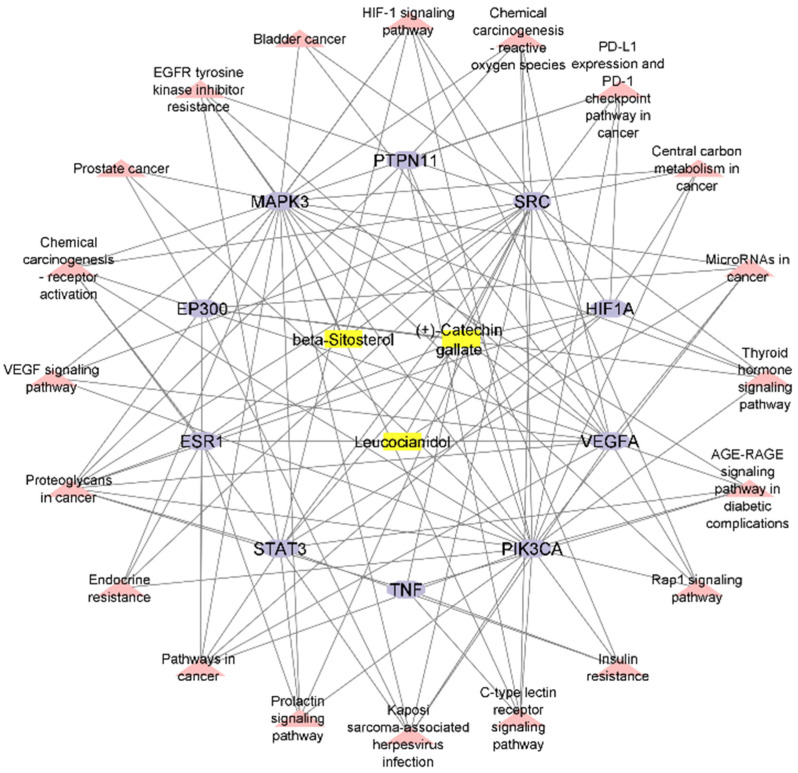
The compound–target–pathway network (yellow: compounds, light purple: hub genes; pink: pathways).

The Cytoscape software was employed for the integration of the compound–target network and the target–pathway network for the construction of the compound–target–pathway network. The Network Analyzer represents 166 edges and 33 nodes in three active phytochemicals, 10 hub genes, and 20 signaling pathways (Figure 7).

### 2.8. Molecular Docking Study

The molecular docking approach was used to explore potential drug candidates to target screened genes from a network pharmacology study for the precise targeting of HCC genes for the treatment of liver cancer. The top three genes (i.e., STAT3, MAPK3, SRC) were selected based on their topological analysis, GO, and KEGG enrichment analyses results. These three genes were at the top of the hub genes, sub-gene clustering, and common in the top 20 GO terms and KEGG pathways. The 3D structures of these target proteins STAT3 (with PDB ID 6TLC), MAPK3 (with PDB ID 6GES), and SRC (with PDB ID 2H8H) were retrieved from the Protein Data Bank in PDB format. The removal of solvent molecules and already bound ligand, energy minimization, and 3D protonation of these proteins were performed using the MOE tool. The 3D structures of the selected phytochemicals were retrieved from the PubChem database in .sdf format.

**Figure 8:**
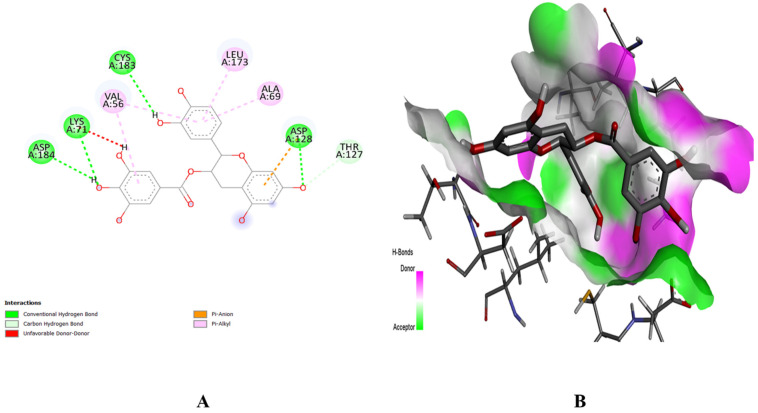
Interaction (**A**) and binding pattern (**B**) of (+)-catechin 3-gallate with active residues of protein MAPK3.

**Figure 9:**
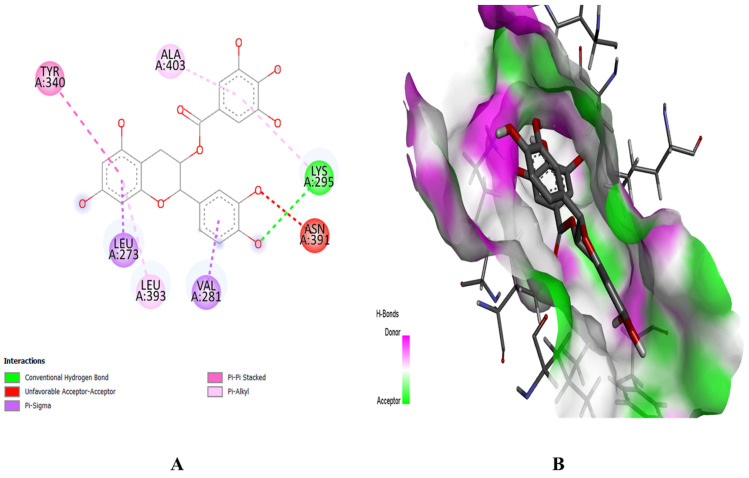
Interaction (**A**) and binding pattern (**B**) of (+)-catechin 3-gallate with active residues of protein SRC.

**Figure 10:**
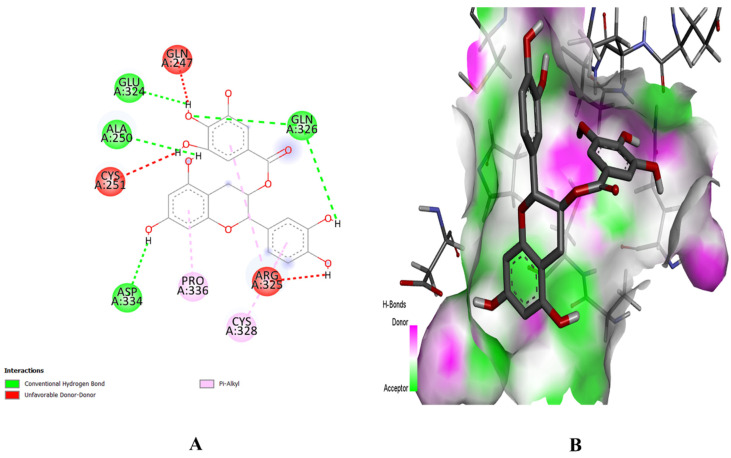
Interaction (**A**) and binding pattern (**B**) of (+)-catechin 3-gallate with active residues of protein STAT3.

The PyRx software was employed for ligand-based molecular docking of the selected compounds with three target proteins. Among the three phytochemicals, (+)-catechin 3-gallate showed the maximum binding score and the strongest interactions with the active residues of all target proteins. The (+)-catechin 3-gallate, with a docking score of −10.2 kcal/mol, interacted with LysA:71, AspA:128, CysA:183, and AspA:184 residues of the binding pocket of MAPK3 via hydrogen bonding (Figure 8). Similarly, (+)-catechin 3-gallate with a docking score of −8.9 kcal/mol interacted with LysA:295 of the SRC protein and with a docking score of −8 kcal/mol interacted with AlaA:250, GluA:324, GlnA:326, and AspA:334 residues of the STAT3 protein via conventional hydrogen bonds (Figure 9 and Figure 10). The other ligands also showed good energy with substantial binding interactions with the active site residues of binding pockets of target proteins (Table 3). BIOVIA Discovery Studio visualizer was used to visualize the ligand–protein 2D interactions and maps.

### 2.9. ADMET Profiling

The ADMET analysis classifies the drug into five parameters (i.e., absorption, distribution, metabolism, excretion, and toxicity). These attributes of a potential drug classify it from the chemical perspective (Table 4). All selected ligands were found to not cross the blood–brain barrier and as being non-Ames toxic. These properties exhibited the potential of these compounds as lead drug candidates for the treatment of HCC.

### 2.10. Cytotoxic Potential of the Best Selected Phytochemicals in HepG2 Cells

**Figure 11:**
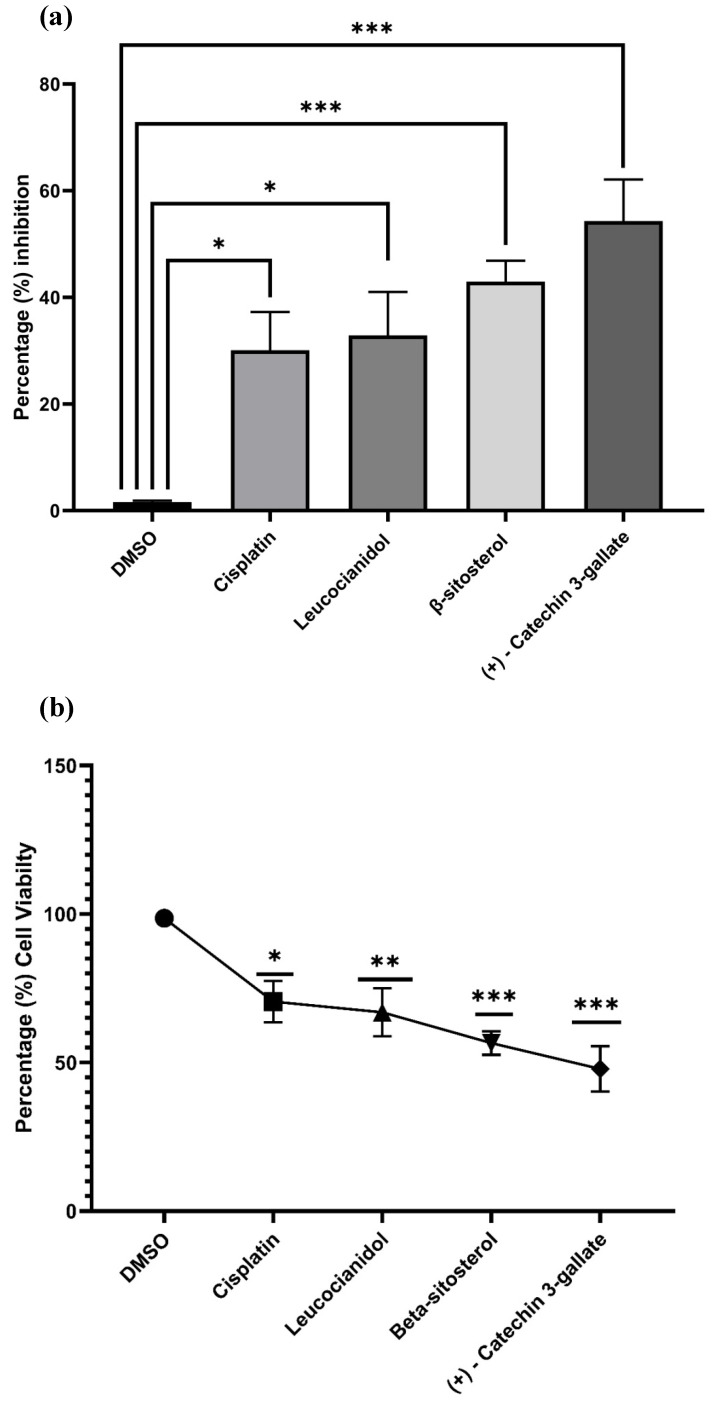
Cytotoxic potential of best selected phytochemicals (i.e., (+)-catechin 3-gallate, β-sitosterol, and leucocianidol). (**a**) Cytotoxicity analysis by MTT assay. The experiment was performed in triplicate and the values are shown as mean ± standard error of mean. (**b**) Percentage of cell viability. If the *p*-value is >0.05, the results were considered statistically significant, represented by * vs. control. ** *p*-value ˃ 0.01 vs. control were considered very significant and *** *p*-value ˃ 0.001 vs. control were considered highly significant. To represent the data, values are presented as mean ± S.E.M.

To examine the cytotoxicity potential of (+)-catechin 3-gallate, β-sitosterol, and leucocianidol in HepG2 cells, the MTT assay was used. Initially, different concentrations of these compounds, including a standard drug cisplatin (i.e., 1.5625, 3.125, 6.25, 12.50, 25, 50, 100, and 200 μg/mL) were applied to HepG2 cells for 24 h and their cytotoxicity potentials were evaluated through an MTT test. The cytotoxicity potential and percent cell viability of control, (+)-catechin 3-gallate, β-sitosterol, and leucocianidol-treated cells are shown in Figure 11. Treatment with (+)-catechin 3-gallate and β-sitosterol significantly (*p* ˂ 0.001) increased the cytotoxicity of HepG2 cells (Table 5). After treatment with 1.56 μg/mL of (+)-catechin 3-gallate and β-sitosterol, a significant inhibitory effect on the viability of HepG2 cells was observed. After 24 h of treatment in HepG2 cells, the IC_50_ values for (+)-catechin 3-gallate, β-sitosterol, and leucocianidol were estimated to be approximately 5.258, 1.784, and 49.94 μg/mL, respectively, compared to cisplatin (i.e., 2.25 μg/mL) (Figure 11).

## 3. Discussion

The pathogenesis of HCC is complex due to sneaking symptoms which make HCC difficult to diagnose. Hepatocarcinogenesis involves the complex cellular dysfunctioning that predominantly transforms into primary liver carcinoma. The lack of predictable biomarkers and continuous resistance make it limited for treatment and sequential therapies. Conventional anticancer therapies have been associated with severe side effects due to lack of diagnosis and selectivity [13]. The vast diversity and low toxicity make plant-derived compounds a more reliable source of drugs compared to synthetic drugs. Plant-derived compounds are much more effective as anticancer agents counter to multiple hallmarks of cancer [14,15]. In most of the cancer pathways, the triggering of apoptosis is an ideal way to induce tumor death and, in this perspective, plant secondary metabolites are reported as natural triggers of apoptosis signaling in different cancers [16].

In the current study, four plants (i.e., *B. ciliate*, *B. pacumbis*, *B. purpurascens*, and *B. stracheyi*) from the Saxifragaceae family have been used due to their anti-inflammatory history as anticancer therapy. The current study is based on previously used *Bergenia* spp. in different cancer therapies. *B. ciliate*, *B. pacumbis*, *B. purpurascens*, and *B. stracheyi* have been reported with great potential as natural sources of antioxidant, anti-inflammatory, and anticancer compounds. Studies have reported the anticancer effects of *Bergenia* spp. on the inhibition of protein kinase and the induction of apoptosis [17]. Similarly, Faheem et al. [18] reported *Bergenia ligulata* silver nanoparticles for arresting p53-mediated mitochondrial apoptosis in breast cancer. The results of their study reveal the potential of BgAgNps as anticancer agents via the cleavage of caspase-3 and downstream targeting of p53 like Bax. In another study, Dulta et al. [19] synthesized zinc oxide NPs from the rhizome extract of *Bergenia ciliate*. The prepared ZnONPs exhibited antibacterial and antioxidant activity against Gram-negative bacterial strains. The ZnONPs with an absorbance band of 340 nm displayed maximum cytotoxic potential in human cervical cancer and human colon cancer cell lines. This indicated the green synthesis of natural NPs to combat with different cancers.

In this study, the overlapping and mostly involving gene(s) in each top signaling pathway for the onset of primary liver carcinoma were selected for precise targeting for the selective treatment of HCC. The ten hub genes with their highest degree ranks in topological analysis and PPIs were further selected for pathway and enrichment analysis. The genes STAT3, MAPK3, SRC, EP300, VEGFA, PIK3CA, TNF, PTPN11, ESR1, and HIF1A were on the top of PPIs and mostly involved in the top twenty GO annotations and KEGG pathways. Among these, the genes STAT3, MAPK3, and SRC were evaluated for HCC treatment. Network pharmacology can be used to create a complex integrated network of compound–target–pathways to understand the complex relation of drugs with their respected targets and signaling mechanisms to manage those specific diseases. These networks are based on targets, biologically active compounds, and biological signaling pathways of the gene that addresses the potential of compounds to tackle the involvement of that specific gene to target that pathway. The selection and screening of targets from the pool of genes make it more reliable to point out the involved gene(s) in common cancer pathways [20].

Signal transducer and activator of transcription 3 (STAT3) modulates chronic inflammation in tumor formulation and mediates interactions between tumor cells and stromal cells. STAT3 belongs to the STAT family with seven members which mediate the signal transduction from the plasma membrane toward the nucleus. The overexpression and ubiquitous activation of STAT3 has been associated with metastasis, immune suppression, and tumor progression in liver cancer. The participation of STAT3 in oncogenesis, angiogenesis, anti-apoptosis, and drug resistance has attracted the attention of researchers as a therapeutic target in liver cancer. Different clinical trials have favored STAT3 gene transcription targeting as effective for cancer treatment [21,22].

Mitogen-activated protein kinase 3 (MAPK3) is a member of the MAP kinase family and called extracellular signal-regulated kinases (ERKs). The overexpression or genetic mutation in the MAPK/ERK signaling cascade has been frequently reported in liver cancer. MAPK/ERK acts as a signaling cascade being involved in differentiation, proliferation, and cell cycle signaling in response to outer signals. Any mutation in this signaling cascade leads to tumor initiation, progression, and modulation of the primary liver tumor, thus leading to HCC [23].

SRC kinase is predominantly involved in multiple cellular signaling pathways including mitochondrial oxidative phosphorylation (OXPHOS). Any abhorrent change in the signaling cascade due to SRC kinase mutation ultimately leads to cancer development and metastasis. The change in OXPHOS signaling and expression has been reported in liver cancer biopsies, which showed the involvement of SRC kinase in HCC [24]. Different clinical specimens have indicated a high level of SRC kinase in liver tumor cells compared to non-tumor cells. The SRC expression has been positively related to tumor stage and metastasis has indicated this kinase as a potential target in liver cancer [25].

The results obtained from network pharmacology were further evaluated through experimental work and the cytotoxic effects of (+)-catechin 3-gallate, β-sitosterol, and leucocianidol were explored. The findings show that (+)-catechin 3-gallate and β-sitosterol have cytotoxic effects on HepG2 cell viability in a dose-dependent manner. In a study, Pal et al. [26] assessed the impact of varying concentrations of epigallocatechin gallate (HGCG) on cell viability in normal fibroblasts and hepatocytes, as well as several hepatoma and colon cancer cell lines including HepG2, Huh7, HLF HCC, HCT-116, HCT-115, and HT29. The results demonstrated a significant decrease in cell viability in all hepatoma and colon cancer cell lines (*p* < 0.01), compared to controls, fibroblasts, and hepatocytes in a dose-dependent manner.

The IC_50_ values for (+)-catechin 3-gallate and β-sitosterol were found to be 5.258 and 1.784 µg/mL, respectively, in the current study. In a study, HepG2 cells were treated with EGCG and metformin and their cell viability exhibited a dose-dependent decrease. The IC_50_ values were determined for each treatment. At 24 h of exposure, the IC_50_ values for EGCG and metformin were calculated to be 31.4 μg/mL and 7.57 μg/mL, respectively [27]. Similarly, Ditty et al. [28] conducted a research to examine the potential cytotoxic effects of β-sitosterol on HepG2 cells using the MTT assay. HepG2 cells were exposed to various concentrations of the compound (i.e., 0.2, 0.4, 0.8, and 1 mM/mL) for a duration of 24 h, and their cytotoxicity was evaluated. The results reveal a significant (*p* < 0.001) induction of dose-dependent cytotoxicity in HepG2 cells following β-sitosterol treatment. The highest level of cytotoxicity was observed at a concentration of 1 mM/mL. At 24 h, the IC_50_ value of β-sitosterol in HepG2 cells was determined to be 0.6 mM/mL. Similarly, Raj [29] also conducted a study to investigate the effect of various concentrations (i.e., 2, 4, 6, 8, and 10 ng/mL) of β-sitosterol-assisted silver nanoparticles (BSS-SNPs) on the morphology of HepG2 cells. The HepG2 cells were treated with BSS-SNPs for a duration of 24 h, and the cytotoxicity was assessed. The results demonstrate a significant and dose-dependent cytotoxicity in HepG2 cells following treatment with BSS-SNPs (*p* < 0.001). The IC_50_ value of BSS-SNPs in HepG2 cells was determined to be 7 ng/mL.

In order to investigate the cytotoxic effects of β-amyrin and β-sitosterol-3-O-glucoside, the MTT cell viability assay was employed on two cancer cell lines (HepG2 and Caco-2) in addition to a non-cancer cell line (HEK293). The results reveal a dose-dependent cytotoxicity of the tested compounds on the cancer cell line. Notably, both β-amyrin and β-sitosterol-3-O-glucoside exhibited selective cytotoxicity toward cancer cells, as indicated by their higher IC_50_ values of 156 and 937 μg/mL, respectively, when tested on the control non-cancer cell line. The Caco-2 cell line demonstrated significant cytotoxic activity when exposed to both compounds, with IC_50_ values of 81 μg/mL for β-amyrin and 54 μg/mL for β-sitosterol-3-O-glucoside [30].

Through network pharmacology, three main targets (i.e., MAPK3, STAT3, and SRC) were evaluated in this study as potential targets for (+)-catechin 3-gallate to treat HCC. These proteins are the main parts of different cancer-leading pathways, which make them perfect targets for the treatment of cancer. The molecular docking has confirmed the potential of (+)-catechin 3-gallate as the lead drug candidate for the treatment of HCC. The minimum energy score has displayed the greater binding affinity of (+)-catechin 3-gallate as a ligand for the selected receptor proteins. This study provides a systemic layout that illustrates the key targets and molecular mechanisms as suggestions or recommendations for a detail study of HCC treatment in the future.

## 4. Materials and Methods

### 4.1. Active Compounds and Targets Prediction

The biologically active compounds from four *Bergenia* spp. (i.e., *B. ciliate*, *B. pacumbis*, *B. purpurascens*, and *B. stracheyi*) were also retrieved from the literature and the publicly available database of Indian Medicinal Plants, Phytochemistry and Therapeutics (IMPPAT) [31]. All predicted compounds were virtually screened for their pharmacokinetic parameters of drug likeness (DL) and oral bioavailability (OB) [32]. Only those compounds were selected for further analysis which met the criteria of DL > 0.18 and OB > 30%. Among different pharmacokinetic parameters, the OB is an important one according to the criteria of absorption, distribution, metabolism, and excretion (ADME). For the determination of the DL index of active compounds, a high OB usually serves as a vital indicator. The compounds with OB ≥ 30% are considered for high oral bioavailability [33]. Similarly, the DL index is also an important tool to screen active compounds rapidly, which serves as a qualitative concept that is applied in drug design for the estimation of drugability of a potential compound. The average DL index in the DrugBank is 0.18 and the compound(s) with ≥0.18 DL index are considered to possess high drugability [34].

### 4.2. Drug Target Profile for Bergenia spp.

The putative gene targets related to selected active phytochemicals were identified and collected by providing SMILES with SwissTargetPrediction tool [35] and STITCH network database [36] by selecting *Homo sapiens* in species option. Protein IDs of each identified protein were aligned with UniProtKB to eliminate duplicates [37].

### 4.3. HCC-Related Target Screening

Different keywords related to the research studies (i.e., hepatocellular carcinoma, liver cancer) were searched to retrieve HCC-related target genes from GeneCard [38] and DisGeNET databases [39] and aligned with UniProtKB IDs to eliminate duplicates. A Ven-diagram was drawn using Jvenn plug-in [40] to illustrate the common overlapping drug-target related genes.

### 4.4. Compound–Target Network

The software Cytoscape 3.9.1 [41] was employed to build the compound–target (CT) network of active constituents of *B. ciliate*, *B. pacumbis*, *B. purpurascens,* and *B. stracheyi* with associated target genes. A plug-in “Network Analyzer” was used to access the topological features of the network.

### 4.5. Protein–Protein Interaction Network

Protein–protein interactions (PPIs) are mandatory to reveal the underlying mechanisms and co-expression of genes [42]. The overlapped genes were imported into the STRING database with a confidence score of 0.7 for PPIs. Multiple protein identifiers were selected with *H. sapiens* as target species to construct the PPI maps. The constructed network was then imported into Cytoscape 3.9.1 software to visualize the topological analysis using Network Analyzer tool. A plug-in “CytoHubba” was used to obtain the hub genes with the highest degree.

### 4.6. Gene Ontology and KEGG Enrichment Analysis

The Gene Ontology (GO) and Kyoto Encyclopedia of Genes and Genomes (KEGG) gene enrichment analysis of selected genes was performed via the DAVID database [43]. DAVID is a functional annotation database which is used to categorize co-occurrence of sets of genes based on cellular component (CC), biological process (BP), and molecular functioning (MF). The KEGG analysis reveals high-level genome mapping that reveals the biological processes and molecular interactions of genes. The probability value of *p* < 0.05 was set to filter the top twenty enriched pathways for pathway–target network construction through Cytoscape 3.9.1 [41]. Bubble plot illustrations for enrichment analysis of GO and KEGG analyses were created through the online tool ShinyGO 0.77 [44].

### 4.7. Compound–Target–Pathway Network

Cytoscape 3.9.1 was used to build the compound–target–pathway (C-T-P) network by integration of compound–target and target–pathway networks. The C-T-P network helps to understand the interrelations of each gene with relevant pathways, which are involved in certain biological and cellular signaling cascades. This networking was used to determine the HCC-associated primary key target associations with the active phytochemicals.

### 4.8. Molecular Docking

The molecular docking study identifies the ligand–target protein interactions to verify the potential of selected ligands as drug candidates [45]. The results of network pharmacology and the potential of selected phytochemicals were further confirmed by the molecular docking approach. The 3D structures of selected receptor proteins were retrieved from the RCSB-PDB database (https://www.rcsb.org/) in .pdb format [46]. Similarly, the chemical structures of selected phytochemicals were retrieved from the PubChem database (https://pubchem.ncbi.nlm.nih.gov/) in .sdf format [47]. The receptor proteins were optimized by removal of ligands, water molecules, and addition of polar hydrogens.

The PyRx software was used for ligand–protein docking to explore the binding patterns of ligands to the active site residues of selected receptor proteins [48]. The docking score was set as selection criterion to choose the best ligands. Finally, the BIOVIA Discovery Studio Visualizer [49] was used to visualize and create 2D/3D figures of interactions and maps of key ligands with target proteins.

### 4.9. ADMET Profiling

The SwissADME [50] and pkCSM [51] freely available online servers were assessed to evaluate the ADMET profiling of selected compounds. The absorption, distribution, metabolism, excretion, and toxicity (ADMET) parameters play a leading role in drug development for finding potential drug candidates [52].

### 4.10. Experimental Study

#### 4.10.1. Hep-G2 Cell Culture

The DMEM (Dulbecco’s Modified Eagle Medium) with 10% fetal bovine serum (FBS) and 100 μL/mL each of streptomycin and penicillin was used to develop the hepatocellular carcinoma cell line Hep-G2. In a CO_2_ incubator, Hep-G2 cells were maintained and allowed to grow at 37 °C with a 5% carbon dioxide source in a moist environment. For treatment purposes, the cells were seeded when sufficient confluence had been reached and 0.25% trypsin-EDTA was added to separate the cells [53].

#### 4.10.2. MTT Cytotoxicity Assay

The MTT assay was used to determine the cytotoxicity-inducing ability of (+)-catechin 3-gallate, β-sitosterol, and leucocianidol. Cisplatin and DMSO were used as a standard drug and control, respectively. Using the MTT assay, the anticancer activities of the best selected phytochemicals were evaluated. For this work, Hep-G2 cells were seeded and planted on 96-well plates. After a 12 h incubation period, different concentrations (1.5625, 3.125, 6.25, 12.50, 25, 50, 100, and 200 μg/mL) of the best selected phytochemicals and cisplatin were delivered to the cancer cells. Following that, cells were treated with 50 µL/mL of MTT solution for 4 h at 37 °C. Then, 0.1% DMSO was also added before aspirating the media. Finally, the ELIZA plate reader was used to note the absorbance at 540 nm [54].

$$1\%=\frac{\left[A540\left(control\right)-A540\left(treated\right)\right]}{A540(control)}\times 100$$

#### 4.10.3. Statistical Analysis

The software Graph Pad Prism 8 was used for the statistical analysis and the significance of the inhibition data was measured using one-way ANOVA. The results with *p*-value > 0.05 were considered as statistically significant [55].

## 5. Conclusions

Hepatocellular carcinoma (HCC) is the most lethal cancer type with malignant tumors mostly reported in Asia and Europe. Despite systemic therapies, there is a continuous increase in death cases due to the limited diagnosis at the advanced stages of cancer and lack of diagnosis biomarkers. Moreover, combinational therapies are associated with adverse side effects that increase the mortality rate with the median postdiagnostic survival of 6–12 after an advanced stage. The network pharmacology approach has pointed out the compound-related targets for the management of HCC. The PPI, GO, and KEGG analyses have explored the top genes involved in the pathogenesis of HCC. The viability on HepG2 cells confirmed the cytotoxic effects of (+)-catechin 3-gallate and β-sitosterol in a dose-dependent manner. In the light of the current results, it has been concluded that *Bergenia* spp. could be used as a natural drug source to treat HCC. Furthermore, clinical and in vivo studies would be required to validate the potential of (+)-catechin gallate, specifically, and β-sitosterol and leucocianidol, generally, as the leading drug candidates for the treatment of HCC in the future.

## Figures and Tables

**Table 1:** Properties of active phytochemicals.

Sr. No.	Compound	Molecular Formula	Oral Bioavailability (>30%)	Drug Likeness (>0.18)	MW (g/mol)	PubChem ID
1	β-Sitosterol	C_29_H_50_O	36.91	0.75	414.79	222284
2	Cianidanol	C_15_H_14_O_6_	54.83	0.24	290.29	9064
3	(+)-catechin gallate	C_22_H_18_O_10_	53.57	0.75	442.4	5276454
4	Leucocianidol	C_15_H_14_O_7_	30.84	0.27	306.29	440833

MW: Molecular weight.

**Table 2:** Degrees of hub gene calculated by Cytoscape.

Sr. No.	Hub Genes	Degrees
1	STAT3	37
2	MAPK3	34
3	SRC	33
4	EP300	25
5	VEGFA	22
6	PIK3CA	22
7	TNF	22
8	PTPN11	21
9	ESR1	19
10	HIF1A	19

**Table 3:** Binding affinities of phytochemicals with target proteins revealed in the molecular docking study.

Sr. No.	Target Protein	PDB ID	UniProt ID	Phytochemical	Binding Energy (kcal/mol)
1	STAT3	6TLC	P40763	(+)-Catechin 3-gallate	−8.0
				β-sitosterol	−7.4
				Leucocianidol	−7.1
2	MAPK3	6GES	P27361	(+)-Catechin 3-Gallate	−10.2
				β-sitosterol	−9.2
				Leucocianidol	−7.4
3	SRC	2H8H	P12931	(+)-Catechin 3-Gallate	−8.9
				β-sitosterol	−8.4
				Leucocianidol	−7.2

**Table 4:** ADMET profiling of lead drug candidates.

ADMET Parameters	Phytochemicals		
	(+)-Catechin 3-Gallate	β-Sitosterol	Leucocianidol
Absorption and distribution			
BBB	No	No	No
Intestinal absorption (human)	62.096%	94.464%	56.712%
PGS	Yes	No	Yes
PGI	No	No	No
Metabolism			
CYP3A4 substrate	No	Yes	No
CYP2D6 substrate	No	No	No
CYP3A4 inhibition	No	No	No
CYP2C9 inhibition	No	No	No
CYP2C19 inhibition	No	No	No
CYP2D6 inhibition	No	No	No
CYP1A2 inhibition	No	No	No
Excretion			
Total Clearance	−0.169 log mL/min/kg	0.628 log mL/min/kg	−0.072 log mL/min/kg
Toxicity			
AMES Toxicity	No	No	No
Hepatotoxicity	No	No	No
Skin Sensitization	No	No	No

Blood–brain barrier (BBB); P-glycoprotein substrate (PGS); P-glycoprotein inhibitor (PGI).

**Table 5:** Comparison of percentage inhibition of control groups and treatments at different concentrations.

Concentration (μg/mL)	DMSO	Catechin	β-Sitosterol	(+)-Catechin 3-Gallate
1.5625	0	7 ^NS^	19.42 ***	16.21 **
3.125	1.3	12 ^NS^	39.3 ****	34.23 ****
6.25	1.7	15 ^NS^	40.2 ****	42.65 ****
12.50	2	29.24 **	41.65 **	55.92 ***
25	2	36.95 ***	49.14 ****	60.05 ****
50	2	41.56 **	64.86 ****	69.6 ****
100	2	43.1 ***	56.29 ****	75.63 ****
200	2	78.4 ****	46.8 ****	80.24 ****
**Concentration (μg/mL)**	**Cisplatin**	**Catechin**	**β-Sitosterol**	**(+)-Catechin 3-Gallate**
1.5625	5.6	7 ^NS^	19.42 **	16.21 *
3.125	10.5	12 ^NS^	39.3 ****	34.23 ****
6.25	13.4	15 ^NS^	40.2 ***	42.65 ***
12.50	27.5	29.24 ^NS^	41.65 ^NS^	55.92 **
25	32.4	36.95 ^NS^	49.14 ^NS^	60.05 **
50	39.5	41.56 ^NS^	64.86 **	69.6 **
100	46.4	43.1 ^NS^	56.29 **	75.63 ***
200	65.4	78.4 ^NS^	46.8 ^NS^	80.24 ****

NS: Non-significant; *: significant; **: very significant; ***, ****: highly significant.

## Data Availability

Data is contained within the article.

## Sample Availability

Sequences and structures used in this study are available from the corresponding author upon request.

## References

[1] Singal A.G., Lampertico P., Nahon P. (2020). Epidemiology and surveillance for hepatocellular carcinoma: New trends. J. Hepatol..

[2] Sakurai T., Kudo M. (2013). Molecular link between liver fibrosis and hepatocellular carcinoma. Liver Cancer.

[3] Sangro B., Sarobe P., Hervás-Stubbs S., Melero I. (2021). Advances in immunotherapy for hepatocellular carcinoma. Nat. Rev. Gastroenterol. Hepatol..

[4] Sayiner M., Golabi P., Younossi Z.M. (2019). Disease burden of hepatocellular carcinoma: A global perspective. Dig. Dis. Sci..

[5] Zhang H., Zhang W., Jiang L., Chen Y. (2022). Recent advances in systemic therapy for hepatocellular carcinoma. Biomark. Res..

[6] Mustafa G., Younas S., Mahrosh H.S., Albeshr M.F., Bhat E.A. (2023). Molecular Docking and Simulation-Binding Analysis of Plant Phytochemicals with the Hepatocellular Carcinoma Targets Epidermal Growth Factor Receptor and Caspase-9. Molecules.

[7] Khalaf A.M., Fuentes D., Morshid A.I., Burke M.R., Kaseb A.O., Hassan M., Hazle J.D., Elsayes K.M. (2018). Role of Wnt/β-catenin signaling in hepatocellular carcinoma, pathogenesis, and clinical significance. J. Hepatocell. Carcinoma.

[8] Llovet J.M., Castet F., Heikenwalder M., Maini M.K., Mazzaferro V., Pinato D.J., Pikarsky E., Zhu A.X., Finn R.S. (2022). Immunotherapies for hepatocellular carcinoma. Nat. Rev. Clin. Oncol..

[9] Dutt R., Garg V., Khatri N., Madan A.K. (2019). Phytochemicals in anticancer drug development. Anti-Cancer Agents Med. Chem..

[10] Koul B., Kumar A., Yadav D., Jin J.-O. (2020). Bergenia genus: Traditional uses, phytochemistry and pharmacology. Molecules.

[11] Ali I., Bibi S., Hussain H., Bano F., Ali S., Khan S.W., Ahmad V.U., Al-Harrasi A. (2014). Biological activities of *Suaeda heterophylla* and *Bergenia stracheyi*. Asian Pac. J. Trop. Dis..

[12] Wang J., Shi J., Jia N., Sun Q. (2022). Network pharmacology analysis reveals neuroprotection of *Gynostemma pentaphyllum* (Thunb.) Makino in Alzheimer’disease. BMC Complement. Med. Ther..

[13] Sharif S., Atta A., Huma T., Shah A.A., Afzal G., Rashid S., Shahid M., Mustafa G. (2018). Anticancer, antithrombotic, antityrosinase, and anti-α-glucosidase activities of selected wild and commercial mushrooms from Pakistan. Food Sci. Nutr..

[14] Mustafa G., Ahmed S., Ahmed N., Jamil A. (2016). Phytochemical and antibacterial activity of some unexplored medicinal plants of Cholistan desert. Pak. J. Bot..

[15] Mustafa G., Arif R., Atta A., Sharif S., Jamil A. (2017). Bioactive compounds from medicinal plants and their importance in drug discovery in Pakistan. Matrix Sci. Pharma.

[16] Ali M., Iqbal R., Safdar M., Murtaza S., Mustafa G., Sajjad M., Bukhari S.A., Huma T. (2021). Antioxidant and antibacterial activities of Artemisia absinthium and Citrus paradisi extracts repress viability of aggressive liver cancer cell line. Mol. Biol. Rep..

[17] Spriha S.E., Rahman S. (2022). In silico evaluation of selected compounds from *Bergenia ciliata* (haw.) sternb against molecular targets of breast cancer. Indian J. Pharm. Educ. Res.

[18] Faheem M.M., Bhagat M., Sharma P., Anand R. (2022). Induction of p53 mediated mitochondrial apoptosis and cell cycle arrest in human breast cancer cells by plant mediated synthesis of silver nanoparticles from Bergenia ligulata (Whole plant). Int. J. Pharm..

[19] Dulta K., Koşarsoy Ağçeli G., Chauhan P., Jasrotia R., Chauhan P. (2021). A novel approach of synthesis zinc oxide nanoparticles by bergenia ciliata rhizome extract: Antibacterial and anticancer potential. J. Inorg. Organomet. Polym. Mater..

[20] Noor F., Tahir ul Qamar M., Ashfaq U.A., Albutti A., Alwashmi A.S., Aljasir M.A. (2022). Network pharmacology approach for medicinal plants: Review and assessment. Pharmaceuticals.

[21] Lee C., Cheung S.T. (2019). STAT3: An emerging therapeutic target for hepatocellular carcinoma. Cancers.

[22] Xu J., Lin H., Wu G., Zhu M., Li M. (2021). IL-6/STAT3 is a promising therapeutic target for hepatocellular carcinoma. Front. Oncol..

[23] Moon H., Ro S.W. (2021). MAPK/ERK signaling pathway in hepatocellular carcinoma. Cancers.

[24] Hunter C.A., Koc H., Koc E.C. (2020). c-Src kinase impairs the expression of mitochondrial OXPHOS complexes in liver cancer. Cell. Signal..

[25] Ren H., Fang J., Ding X., Chen Q. (2016). Role and inhibition of Src signaling in the progression of liver cancer. Open Life Sci..

[26] Pal D., Sur S., Roy R., Mandal S., Kumar Panda C. (2019). Epigallocatechin gallate in combination with eugenol or amarogentin shows synergistic chemotherapeutic potential in cervical cancer cell line. J. Cell. Physiol..

[27] Sabry D., Abdelaleem O.O., El Amin Ali A.M., Mohammed R.A., Abdel-Hameed N.D., Hassouna A., Khalifa W.A. (2019). Anti-proliferative and anti-apoptotic potential effects of epigallocatechin-3-gallate and/or metformin on hepatocellular carcinoma cells: In vitro study. Mol. Biol. Rep..

[28] Ditty M.J., Ezhilarasan D. (2021). β-sitosterol induces reactive oxygen species-mediated apoptosis in human hepatocellular carcinoma cell line. Avicenna J. Phytomedicine.

[29] Raj R.K. (2020). β-Sitosterol-assisted silver nanoparticles activates Nrf2 and triggers mitochondrial apoptosis via oxidative stress in human hepatocellular cancer cell line. J. Biomed. Mater. Res. Part A.

[30] Maiyoa F., Moodley R., Singh M. (2016). Phytochemistry, cytotoxicity and apoptosis studies of β-sitosterol-3-oglucoside and β-amyrin from Prunus africana. Afr. J. Tradit. Complement. Altern. Med..

[31] Vivek-Ananth R., Mohanraj K., Sahoo A.K., Samal A. (2023). IMPPAT 2.0: An enhanced and expanded phytochemical atlas of Indian medicinal plants. ACS Omega.

[32] Ru J., Li P., Wang J., Zhou W., Li B., Huang C., Li P., Guo Z., Tao W., Yang Y. (2014). TCMSP: A database of systems pharmacology for drug discovery from herbal medicines. J. Cheminform..

[33] Guo W., Huang J., Wang N., Tan H.-Y., Cheung F., Chen F., Feng Y. (2019). Integrating network pharmacology and pharmacological evaluation for deciphering the action mechanism of herbal formula zuojin pill in suppressing hepatocellular carcinoma. Front. Pharmacol..

[34] Tao W., Xu X., Wang X., Li B., Wang Y., Li Y., Yang L. (2013). Network pharmacology-based prediction of the active ingredients and potential targets of Chinese herbal Radix Curcumae formula for application to cardiovascular disease. J. Ethnopharmacol..

[35] Daina A., Michielin O., Zoete V. (2019). SwissTargetPrediction: Updated data and new features for efficient prediction of protein targets of small molecules. Nucleic Acids Res..

[36] Szklarczyk D., Santos A., Von Mering C., Jensen L.J., Bork P., Kuhn M. (2016). STITCH 5: Augmenting protein–chemical interaction networks with tissue and affinity data. Nucleic Acids Res..

[37] The UniProt Consortium (2021). UniProt: The universal protein knowledgebase in 2021. Nucleic Acids Res..

[38] Safran M., Rosen N., Twik M., BarShir R., Stein T.I., Dahary D., Fishilevich S., Lancet D. (2021). The genecards suite. Practical Guide to Life Science Databases.

[39] Piñero J., Ramírez-Anguita J.M., Saüch-Pitarch J., Ronzano F., Centeno E., Sanz F., Furlong L.I. (2020). The DisGeNET knowledge platform for disease genomics: 2019 update. Nucleic Acids Res..

[40] Bardou P., Mariette J., Escudié F., Djemiel C., Klopp C. (2014). jvenn: An interactive Venn diagram viewer. BMC Bioinform..

[41] Shannon P., Markiel A., Ozier O., Baliga N.S., Wang J.T., Ramage D., Amin N., Schwikowski B., Ideker T. (2003). Cytoscape: A software environment for integrated models of biomolecular interaction networks. Genome Res..

[42] Arif R., Zia M.A., Mustafa G. (2021). Structural and functional annotation of napin-like protein from momordica charantia to explore its medicinal importance. Biochem. Genet..

[43] Sherman B.T., Hao M., Qiu J., Jiao X., Baseler M.W., Lane H.C., Imamichi T., Chang W. (2022). DAVID: A web server for functional enrichment analysis and functional annotation of gene lists (2021 update). Nucleic Acids Res..

[44] Ge S.X., Jung D., Yao R. (2020). ShinyGO: A graphical gene-set enrichment tool for animals and plants. Bioinformatics.

[45] Mustafa G., Mahrosh H.S., Attique S.A., Arif R., Farah M.A., Al-Anazi K.M., Ali S. (2023). Identification of Plant Peptides as Novel Inhibitors of Orthohepevirus A (HEV) Capsid Protein by Virtual Screening. Molecules.

[46] Bittrich S., Rose Y., Segura J., Lowe R., Westbrook J.D., Duarte J.M., Burley S.K. (2022). RCSB Protein Data Bank: Improved annotation, search and visualization of membrane protein structures archived in the PDB. Bioinformatics.

[47] Kim S., Chen J., Cheng T., Gindulyte A., He J., He S., Li Q., Shoemaker B.A., Thiessen P.A., Yu B. (2019). PubChem 2019 update: Improved access to chemical data. Nucleic Acids Res..

[48] Dallakyan S., Olson A.J., Hempel J., Williams C., Hong C. (2015). Small-molecule library screening by docking with PyRx. Chemical Biology. Methods in Molecular Biology.

[49] Sharma S., Sharma A., Gupta U. (2021). Molecular Docking studies on the Anti-fungal activity of *Allium sativum* (Garlic) against Mucormycosis (black fungus) by BIOVIA discovery studio visualizer 21.1. 0.0. Res. Sq..

[50] Daina A., Michielin O., Zoete V. (2017). SwissADME: A free web tool to evaluate pharmacokinetics, drug-likeness and medicinal chemistry friendliness of small molecules. Sci. Rep..

[51] Pires D.E., Blundell T.L., Ascher D.B. (2015). pkCSM: Predicting small-molecule pharmacokinetic and toxicity properties using graph-based signatures. J. Med. Chem..

[52] Azeem M., Mustafa G., Mahrosh H.S. (2022). Virtual screening of phytochemicals by targeting multiple proteins of severe acute respiratory syndrome coronavirus 2: Molecular docking and molecular dynamics simulation studies. Int. J. Immunopathol. Pharmacol..

[53] Rasul A., Riaz A., Wei W., Sarfraz I., Hassan M., Li J., Asif F., Adem Ş., Bukhari S.A., Asrar M. (2021). *Mangifera indica* extracts as novel PKM2 inhibitors for treatment of triple negative breast cancer. BioMed Res. Int..

[54] Zara R., Rasul A., Sultana T., Jabeen F., Selamoglu Z. (2022). Identification of *Macrolepiota procera* extract as a novel G6PD inhibitor for the treatment of lung cancer. Saudi J. Biol. Sci..

[55] Abdel-Hamid N.M., EL-Gharieb M.S., El-Senduny F.F., Alnakib N.A.-A. (2022). Effect of Doxorubicin and Cisplatin on Alpha-fetoprotein levels in Hepatocellular Carcinoma Cell lines. Alfarama J. Basic Appl. Sci..

